# Study on the 3D printability of poly(3-hydroxybutyrate-*co*-3-hydroxyvalerate)/poly(lactic acid) blends with chain extender using fused filament fabrication

**DOI:** 10.1038/s41598-020-68331-5

**Published:** 2020-07-16

**Authors:** Miguel A. Vigil Fuentes, Suman Thakur, Feng Wu, Manjusri Misra, Stefano Gregori, Amar K. Mohanty

**Affiliations:** 1 School of Engineering, University of Guelph, Guelph, ON N1G 2W1 Canada; 2 Departmet of Plant Agriculture, Crop Science Building, Bioproducts Discovery and Development Center, University of Guelph, Guelph, ON N1G 2W1 Canada

**Keywords:** Polymers, Design, synthesis and processing, Biomedical materials

## Abstract

In this study, the 3D printability of a series of poly(3-hydroxybutyrate-*co*-3-hydroxyvalerate) (PHBV)/poly(lactic acid) (PLA) blends were investigated using fused filament fabrication (FFF). The studied blends suffered from poor 3D printability due to differences in compatibility and low thermal resistance. These shortcomings were addressed by incorporating a functionalized styrene-acrylate copolymer with oxirane moieties as a chain extender (CE). To enhance mechanical properties, the synergistic effect of 3D printing parameters such as printing temperature and speed, layer thickness and bed temperature were explored. Rheological analysis showed improvement in the 3D printability of PHBV:PLA:CE blend by allowing a higher printing temperature (220 °C) and sufficient printing speed (45 mm s^−1^). The surface morphology of fractured tensile specimens showed good bonding between layers when a bed temperature of 60 °C was used and a layer thickness of 0.25 mm was designed. The optimized printing samples shown higher storage modulus and strength, resulting in stiffer and stronger parts. The crystallinity, morphology and performance of the 3D printed products were correlated to share key methods to improve the 3D printability of PHBV:PLA based blends which may be implemented in other biopolymer blends, and further highlight how process parameters enhance the mechanical performance of 3D printed products.

## Introduction

Conventional sources of energy such as fossil fuels are becoming less sustainable and reliable due to our limited natural resources^1^. The development of greener materials with at least similar mechanical properties to conventional feedstock is of importance for their successful implementation in manufacturing sectors such as additive manufacturing (AM)^2^. Fused filament fabrication (FFF) is an AM technology in which thermoplastic materials are melted and deposited layer by layer onto a free surface platform^3^. FFF allows freedom of design, which facilitates prototyping and customization of products in a timely and cost-effective manner. Due to the nature of FFF processing, material properties are highly anisotropic and research on materials, as well as printing parameters such as layer width, thickness, raster angle and air gap between layers, is vital for the optimization of fabricated products^3–6^. Consequently, previous researchers have determined that layer orientation of 0° raster angle in the axial load direction results in higher tensile properties, while products with build orientation angles of ± 45° have improved impact strength and less warping^7–9^.

Polyhydroxyalkanoates (PHAs) are gaining immense attention in the field of biopolymers due to their inherent nature such as biodegradability, biocompatibility and promise for use in a range of applications, especially in the biomedical industry^10,11^. In addition, PHAs are reducing the high dependency on petroleum-based materials. Among PHAs, poly(3-hydroxybutyrate-*co*-3-hydroxyvalerate) (PHBV) is currently one of the most widely produced and commercialized bioplastics. PHBV is an aliphatic thermoplastic material synthesized by a range of different microorganisms as intracellular inclusions in the presence of carbon source and under limited growth conditions^10,11^. PHBV has been used in various processing methods such as injection molding, solvent casting and electrospinning^11^. However, research of PHBV in FFF remains to be explored.

On the other hand, PLA is a suitable and widely studied thermoplastic for FFF based three-dimensional (3D) printing. In particular, the 3D printability of PLA has been widely explored in FFF using different 3D printers, additives or biopolymer blends^9,12,13^. Benwood et al*.*^9^ focused on improving the mechanical properties of PLA by optimizing 3D printing parameters. It was concluded that using bed surface temperatures (90 °C) higher than the glass transition temperature of PLA resulted in increased crystallinity and impact strength of the samples. Tymrak et al*.*^12^ explored the 3D printability of PLA using an open source 3D printer. The appropriate selection of printing parameters resulted in printed samples with similar mechanical properties when compared to other commercial 3D printers. Ou-Yang et al*.*^13^ studied different PLA and poly(butylene succinate) (PBS) based blends, and improved product distortion by using 40% or more PLA content^13^.

In general, 3D printing of semi-crystalline polymers with a high degree of crystallinity often results in poor product dimensional accuracy or warpage due to shrinkage and residual stresses^14,15^. Jin et al*.*^14^ investigated the warpage and interlayer bonding of polypropylene (PP) using short isometric filaments**.** They compared various PP polymer grades and obtained a correlation of increase in product deformation and stiffness to PP grades with a higher degree of crystallinity. In addition, the fabrication of filaments with isometric dimensions was of utmost importance during this study for consistency of 3D printed samples^14^. PHBV is a semi-crystalline thermoplastic material with a high degree of crystallinity and narrow processing window because of the small difference between melting and thermal degradation temperatures^11^. As a result, blending PHBV with other biopolymers and polymer agents has been used as means to improve these limitations.

The use of chain extenders (CE) has been investigated in melt extrusion to increase molecular weight and thermal stability of various polymers^16,17^. Duangphet et al*.*^16^ found that the addition of a CE with functionalized styrene-acrylate copolymer with oxirane moieties to PHBV improved its thermal stability and complex viscosity. In addition, a reduction in the polymer chain mobility resulted in a lower degree of crystallinity and crystal imperfections due to the cross-linking or the branching effect of the CE^16^. Corre et al*.*^17^ found that this CE reacted effectively with PLA. Addition of 0.9 wt% CE increased the complex viscosity of PLA approximately 30 times. It is hypothesized that the incorporation of appropriate amounts of chain extender along with a biopolymer such as PLA can be used to enhance the 3D printability of PHBV by improving its rheological and thermal properties, as previous study shown that the thermal stability of PHBV can be improved through blending with PLA^18^.

Recent studies have explored the 3D printability of PHAs and PLA using FFF^19,20^. Menčík et al.^19^ explored the 3D printability of poly(3-hydroxybutyrate) (PHB):PLA based blends using four commercial plasticizers. They observed improvement in dimensional stability and reduced warping by incorporating 15 wt% plasticizer. This resulted from the plasticizing effect on PHB based blends. Another study investigated the effect of bed temperature on dumbbell shaped samples printed in vertical and horizontal raster angle directions using commercial PLA:PHAs filaments^20^. It was found that the contact time as well as the surface contact area had an effect on the crystalline phase and biodegradability of the blends. However, the effect of bed temperature on the mechanical properties of the blends was not studied. To our best knowledge, no current research has investigated the 3D printability of PHBV:PLA based blends with chain extender or the effect 3D printing processing parameters have on the mechanical properties of PHBV:PLA blends.

The main objectives of this study were to improve the 3D printability and enhance the mechanical properties of PHBV:PLA based blends using a CE. With CE added, PHBV:PLA blend filaments were extruded at high amount of PHBV (60 wt%) without melt fracture. In addition, the blends with CE were successfully 3D printed and the properties were evaluated. Incorporation of CE assisted to achieve appropriate rheological properties and reach optimal printing temperature as well as speed for preventing the thermal degradation of PHBV during printing. Thermal properties, polarized microscopy and surface morphology were used to evaluate the material properties of the selected PHBV:PLA:CE blends. The use of adequate CE content and the synergistic effect of 3D printing parameters enhanced the mechanical properties of the blends. Dynamic thermo-mechanical and mechanical analysis was used to evaluate part performance. Finally, the surface morphology of tensile fractured samples was used to further assess the bonding between layers after mechanical failure. This provided insight into the diffusion-based fusion behavior of the products during the printing process. The optimized 3D printed biodegradable PHBV:PLA products suggests potential use in biomedical applications for its high mechanical strength and good biocompatibility.

## Results and discussion

### Optimization of 3D printing parameters

**Figure 1:**
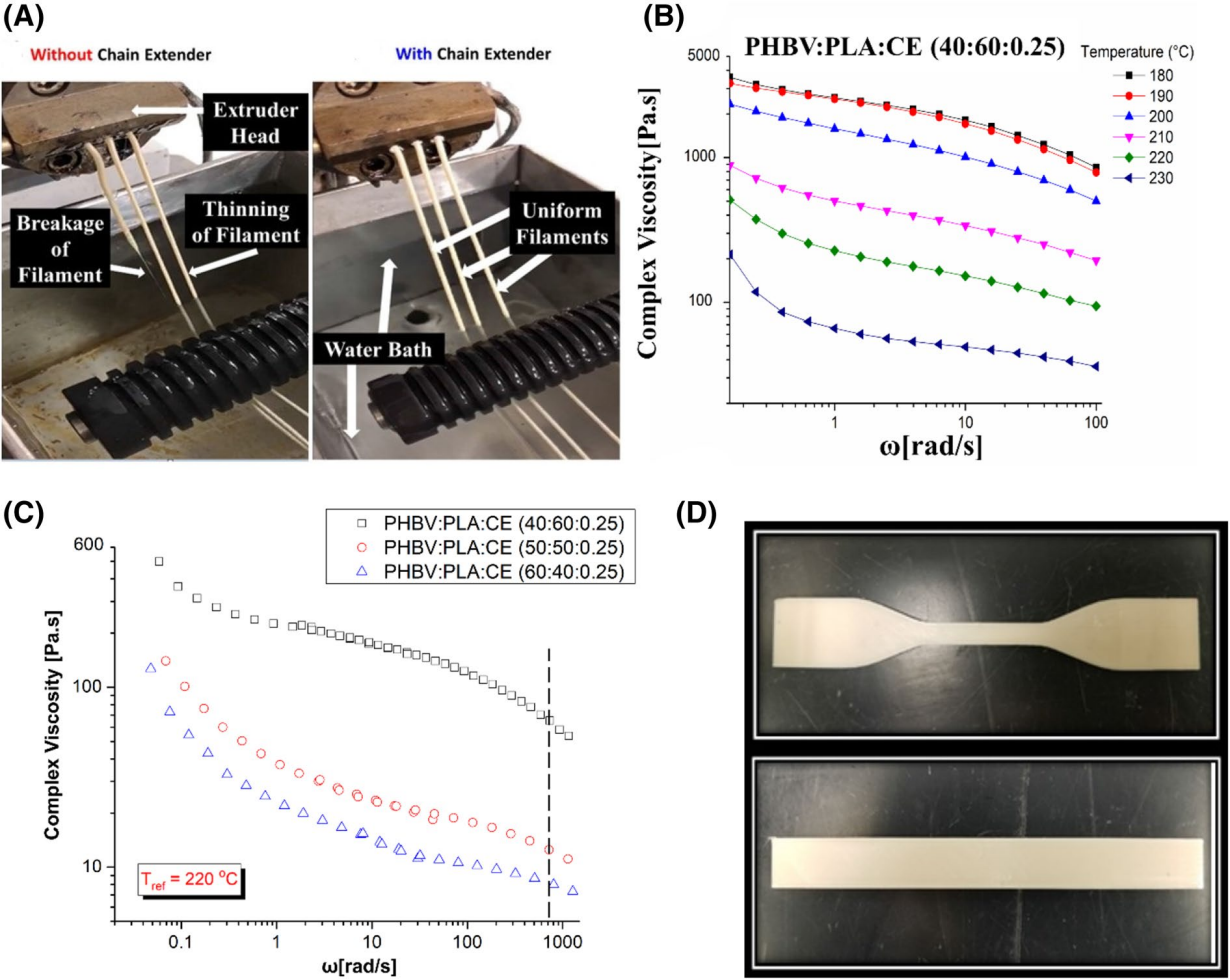
The optimization of 3D printing parameter and 3D printed samples: (**A**) the effect of chain extender on the melt-extrusion filament performance (PHBV:PLA (40:60) blend); (**B**) the effect of temperature on the complex viscosity of PHBV:PLA:CE (40:60:0.25) blend; (**C**) the effect of printing speed on the complex viscosity of PHBV:PLA:CE blends; (**D**) the 3D printed PHBV:PLA:CE (40:60:0.25) tensile and flexural bar at optimized temperature and printing speed. The (**B**,**C**) were generated from Origin 8, OriginLab, (**A**,**D**) were taken by the author.

As a stretching process, successful 3D printing via FFF method requires consistent filaments with high melt strength so that no melt fracture occurs during printing. For example, Lau et al*.*^21^ investigated the effect of melt strength for the thermoforming of polypropylene (PP). It was found that an increase in the melt strength of PP minimized sagging problems during thermoforming processes^21^. Similar aspects were found in this work. The filament performance of the PHBV:PLA (40:60) blends with and without chain extender (CE) after melt-extrusion was shown in Fig. 1A. PHBV: PLA based blends with weight ratios of (60:40), (50:50) and (40:60) were attempted, however, due to the immiscibility and absence of chain entanglement of PHBV and PLA^22,23^, the prepared blends exhibited extremely low melt strength, resulting in 3D printing failure. In addition to the low melt strength, the poor thermal stability of PHBV also led to serious thermal degradation of the blends during the printing process. Consequently, a chain extender (CE) was used as an effective method to improve the melt strength of the blends by fostering the entanglement of polymeric chains and limiting the thermal degradation of the chains during the melt process. As a result, consistent filaments were successfully extruded and the processing window for 3D printing was widened such that the blends could be printed at higher temperatures. Based on preliminary evaluation and existing research^16,17,24,25^, as well as taking the biocompatibility into consideration, a low amount of CE (0.25 parts per hundred—phr) was selected for this study.

The printing temperature and speed directly influence the surface finish and properties of the samples. This is due to the viscosity will be changed at different printing temperature and speed. To evaluate the influence of temperature on the viscosity, the rheological testing of the PHBV:PLA:CE (60:40:0.25) was given in Fig. 1B and the complex viscosity of other two blends was given in Supplementary Fig. S1. At a lower temperature, for example 200 °C, the viscosity of the blends was too high when extruded from the nozzle, resulting in failure during 3D printing. Qahtani et al*.*^26^ reported delamination and poor object fabrication for PLA:BioPBS 3D printed products due to high viscosity values. Consequently, good adhesion of 3D printed products was achieved by using higher printing temperatures. Increasing the printing temperature beyond 200 °C was of concern as PHBV might degrade in high temperature^27^. Capone et al*.*^28^ investigated the degradation effects of polymethyl-methacrylate and polystyrene at various processing temperatures and mechanical stresses. Jagenteufel et al*.*^29^ explored thermal degradation of acrylonitrile butadiene styrene (ABS) using FFF. They concluded^28,29^ that thermal degradation was negligible due to the short residence time of the polymer during extrusion. However, a printing temperature of 230 °C led to thermal degradation due to the nozzle heat dissipation over existing sublayers, resulting in printing failure. The viscosity of the material was decreased to a suitable value without thermal degradation of the material using a printing temperature of 220 °C. The complex viscosity of the blends decreased with increasing PHBV content, resulting from the lower viscosity of PHBV compared to that of PLA, and higher reactivity between the PLA and CE^16,17^, as seen in Supplementary Fig. S2A. Moreover, all blends exhibited viscoelastic liquid behavior at all ranges since the loss modulus was greater than the storage modulus, as seen in Supplementary Fig. S2B,C. This is crucial in FFF for successful 3D printability as continuous flowability of the polymer is required at the time of extrusion.

In addition to the printing temperature, printing speed was also important in 3D printing due to the exposure time (time which polymer is subject to heating in the chamber, where over exposure leads to degradation) and was controlled by the 3D printing speed. Therefore, different speeds were used (up to 55 mm s^−1^) and it was found that 45 mm s^−1^ was the best printing speed to obtain perfect printed products. By using time–temperature superposition (TTS) rheological curves, the complex viscosity of the blends at high printing speed (45 mm s^−1^) can be obtained, as shown in Fig. 1C. The protocol for the preparation of the TTS master curve is summarized in the Experimental Section. The complex viscosity was found to be in the range between 8 and 80 Pa s. To successfully 3D print the PHBV:PLA blends, it is suggested that the complex viscosity of those blends is within this range. In addition, Seppala et al*.*^30^ modeled the weld formation between layers and found that weld formation was dependent on the temperatures of the sublayer and the layer being printed. Lower zero-shear viscosity values can improve weld formation between layers due to good diffusion-based fusion between the newly printed layer and sublayer^26,30^.

In summary, the printing parameters for PHBV:PLA blends are mainly controlled by the viscosity and thermal stability of the materials. It appeared from the present experiments that high temperature (220 °C) and fast speed (45 mm s^−1^) should be adopted to decrease the viscosity to 8–80 Pa s but avoid thermal degradation during the PHBV:PLA:CE 3D printing. Successful 3D printing of all PHBV:PLA:CE based blends was achieved using a printing temperature of 220 °C and printing speed of 45 mm s^−1^, as shown by the tensile and flexural bar in Fig. 1D.

### Co-relationship discussion on bed temperature and crystallization

**Figure 2:**
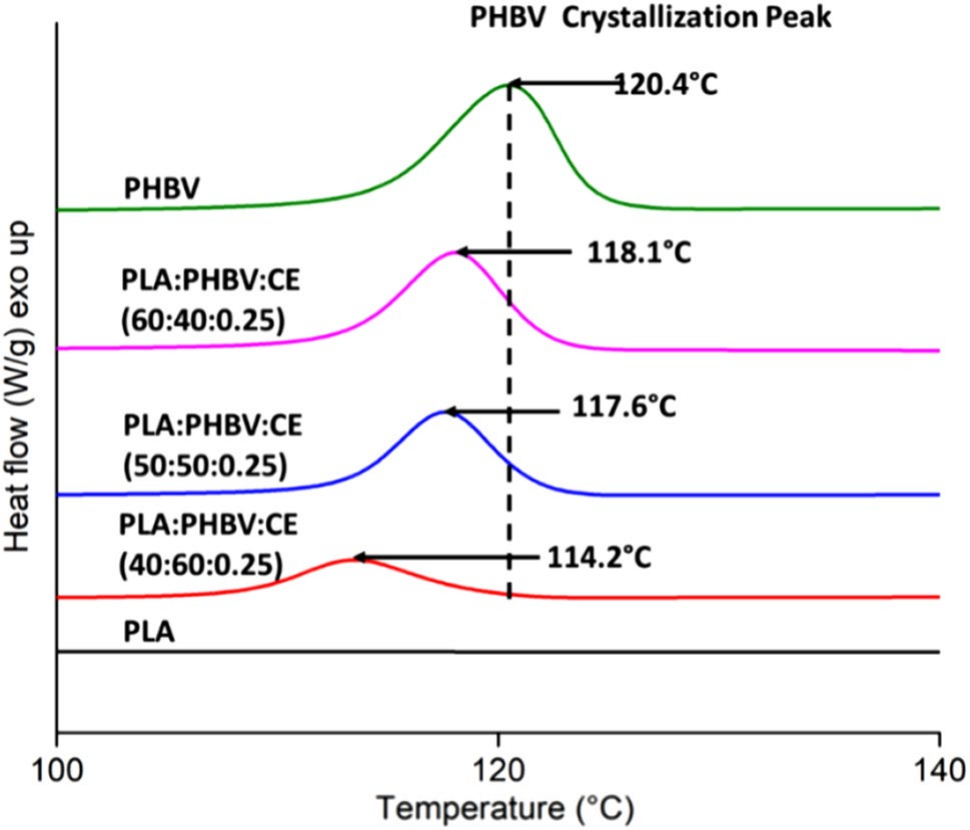
Thermal analysis for the cooling cycle of PLA, PHBV and PHBV:PLA:CE based blend. The figures were generated from Origin 8, OriginLab.

The thermal properties of the blends were studied using differential scanning calorimetry (DSC). The second heating and cooling cycles were of interest to evaluate thermal properties such as *T*_g_, *T*_m_, degree of crystallinity (*X*_c_) and cold crystallization (*T*_cc_) temperature of the neat polymers in comparison with the fabricated blends. The thermal properties of the second heating cycle are summarized in Table 1. In particular, the *T*_g_ of PLA was of importance to select an appropriate bed temperature and improve bed adhesion. In FFF, bed temperatures slightly above the *T*_g_ of the polymer can be used to enhance interfacial adhesion between the printed product and the bed surface^31,32^. The *T*_g_ of PLA in the blends was measured by DSC to be between 58 and 59 °C. This was expected as PLA and PHBV have been previously reported to be immiscible^21,22^. The *T*_g_ of PHBV was detected for the PHBV:PLA:CE (40:60:0.25) wt% based blend at approximately 2.2 °C. Other studies have reported the *T*_g_ of PHBV for similar weight ratios between 0 and 3 °C^21,22^. A decrease in the degree of crystallinity of PHBV was observed with increasing PLA content. Similarly, a shift in the *T*_cc_ peak of PHBV was observed from 120.4 to 114.2 °C with increasing PLA content, as seen in Fig. 2. This was expected as blending PHBV with PLA in the presence of a CE resulted in chemical bonding between the polymers hindering their crystallization. In addition, a reduction in the melting enthalpy of PLA was observed for all blend compositions. The reduction in the melting enthalpy of PLA was attributed to the CE branching or crosslinking effect.

**Table 1:** Thermal properties of second heating cycle using DSC of the PHBV: PLA: CE blend.

Materials	*T*_g_ (°C)		*T*_m_ (°C)		*X*_c_ (%)	
PHBV:PLA:CE	PLA	PHBV	PLA	PHBV	PLA	PHBV
0:100:0	61.1 ± 0.1	–	148.8 ± 0.1	–	6.3 ± 0.9	–
100:0:0	–	–	–	173.4 ± 0.6	–	60.1 ± 0.2
60:40:0.25	58.8 ± 0.4	–	148.8 ± 0.2	172.0 ± 0.0	1.8 ± 0.2	56.1 ± 0.4
50:50:0.25	58.6 ± 0.5	–	145.6 ± 0.3	171.5 ± 0.1	1.3 ± 0.6	53.4 ± 0.6
40:60:0.25	59.5 ± 0.1	2.2 ± 0.2	149.9 ± 0.3	171.1 ± 0.4	2.5 ± 0.5	39.1 ± 5.0

**Figure 3:**
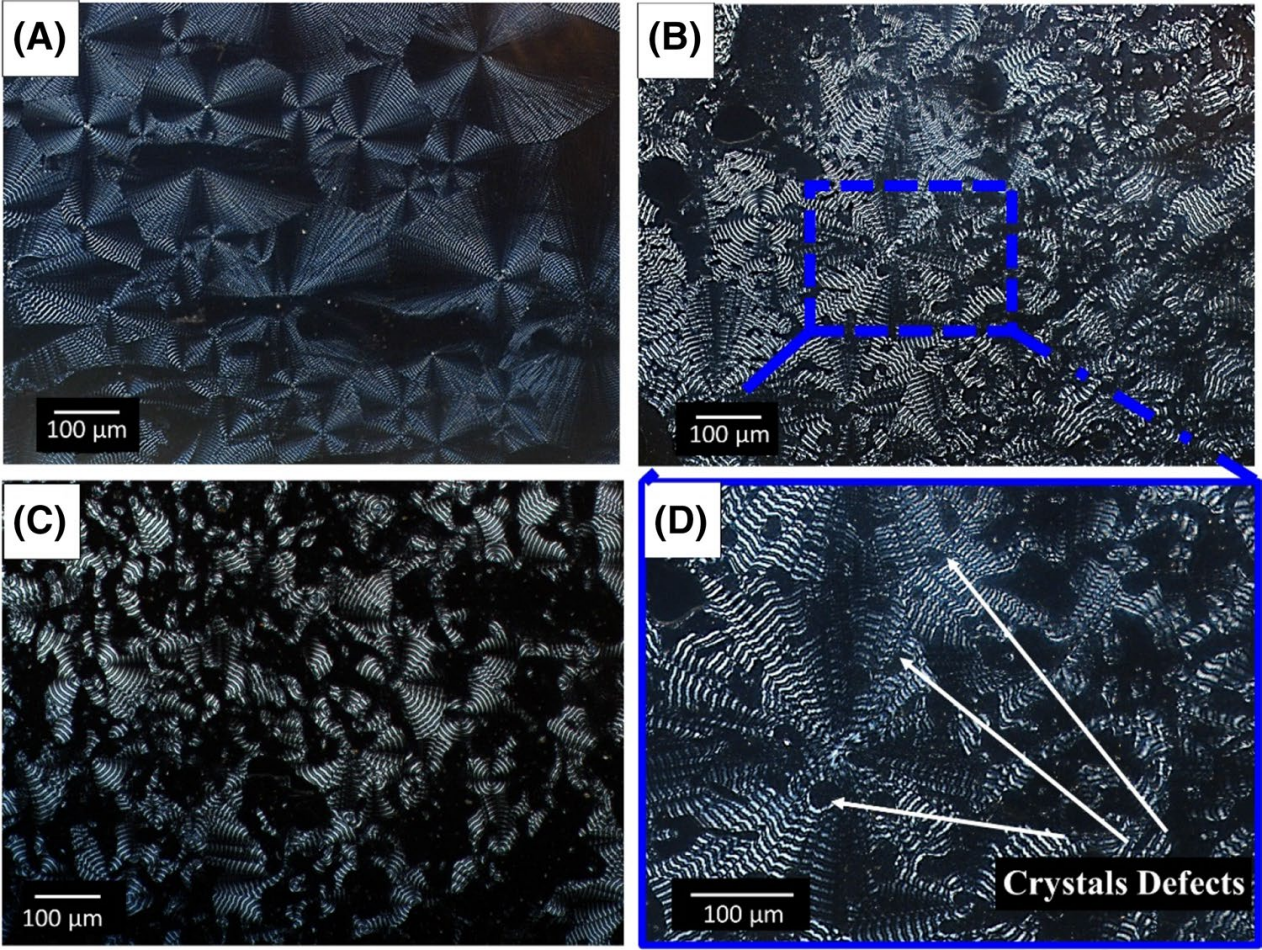
Crystal morphology of PHBV:PLA:CE based blends after 30 min at 60 °C (**A**) (60:40:0.25) wt% using × 10 magnification, (**B**) (50:50:0.25) wt% using × 10 magnification, (**C**) (40:60:0.25) wt% using × 10 magnification and (**D**) (50:50:0.25) wt% using × 20 magnification. The photos were generated from NIS-Elements BR3.2 64-bit.

The morphology of crystals was investigated during an isotherm at 60 °C. The selection of this parameter was based on the bed temperature chosen for 3D printing. No crystal growth was observed for neat PLA under this condition. This was expected as PLA has a *T*_g_ of approximately 60 °C and a low degree of crystallinity. For PHBV, a characteristic Maltese cross pattern was observed, which is typical of semi-crystalline polymers due to the birefringence of the 3D spherulites. Similar observations have been reported for the crystal structure of PHBV by other researchers^22,33^. PHBV had the largest spherulite radius of all materials examined. Supplementary Figure S3 shows the morphology of PHBV crystals at several time intervals. Complete spherulite growth was observed for PHBV after 8 min. With increasing PLA content in the PHBV:PLA:CE blend, local absence of birefringence was observed resulting in what is called in the literature ‘crystal defects’. The crystal morphology of PHBV:PLA:CE (60:40:0.25), (50:50:0.25) and (40:60:0.25) wt% based blends and PHBV crystal defects can be seen in Fig. 3. The spherulite radius length decreased with increasing PLA content. This correlates with the decrease in the degree of crystallinity of PHBV observed by thermal analysis. A previous study by Duangphet et al*.*^33^ reported the effect of CE on PHBV. It was concluded that using 1 phr CE content did not affect its crystal morphology^33^. It is assumed that the incorporation of PLA and CE to PHBV contributed to the reduction and disordering of its chain structure, leading to crystal defects.

### Dynamic mechanical analysis of 3D printing samples

**Figure 4:**
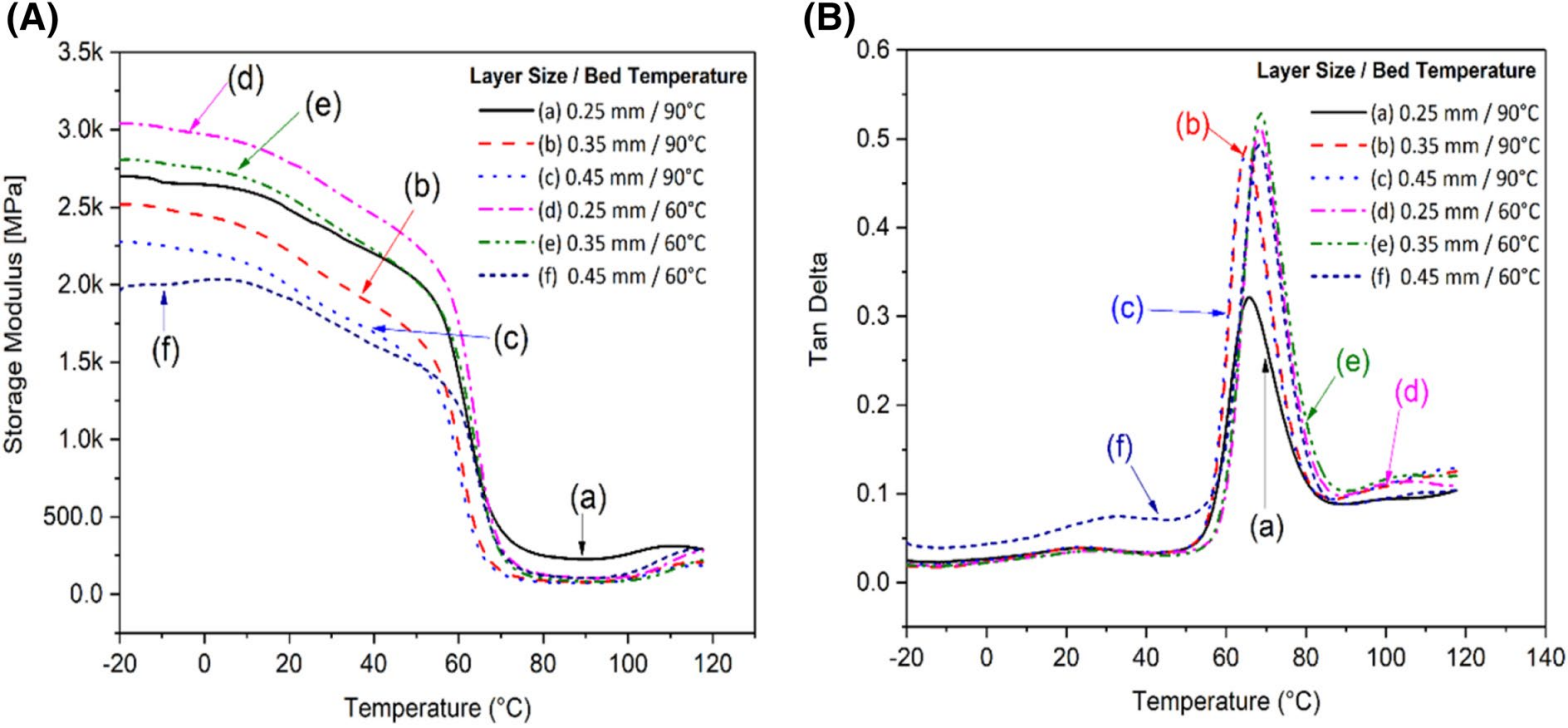
DMA plot of (**A**) storage modulus, and (**B**) tan δ for different layer thicknesses and bed temperatures of PHBV:PLA:CE (40:60:0.25) wt% blend. The figures were generated from Origin 8, OriginLab.

The dynamic mechanical behaviour of the 3D printed parts was investigated by conducting dynamic mechanical analysis (DMA) from − 20 to 120 °C. The storage modulus and tan delta (δ) for different layer thicknesses and bed temperatures for PHBV:PLA:CE (40:60:025) wt% blend can be found in Fig. 4. The tan δ peak corresponds to the ratio of energy dissipated and stored per deformation cycle and is often used to measure the *T*_g_ of polymers. The tan δ peak for PHBV was found to be between 22 to 27 °C, while for PLA between it was 64 to 68 °C. The layer thickness did not show a significant influence while an increase of the bed temperature resulted in a slight shift of *T*_g_ to higher temperatures. In a previous work, Nanda et al*.*^34^ reported the storage modulus for neat PLA and PHBV as 3.5 GPa and 1.8 GPa, respectively, using injection molding. The highest storage modulus was obtained for samples with a layer thickness of 0.25 mm and a bed temperature of 60 °C at approximately 2.7 GPa. Using a design of experiment approach, Shubham et al*.*^6^ reported better dynamic mechanical properties for samples with a layer thickness of 0.20 mm due to enhanced structural integrity. The layer thicknesses explored were 0.10 mm, 0.15 mm, 0.20 mm and 0.25 mm^6^. In this study 3D printed products with 0.25 mm layer thickness showed higher storage modulus of the samples due to having more layers per sample and smaller cell size between layers resulting in stiffer objects. The lowest storage modulus was observed in samples with a layer thickness of 0.45 mm and bed temperature of 90 °C at approximately 2.0 GPa.

### Morphological characterization of 3D printing parameters

**Figure 5:**
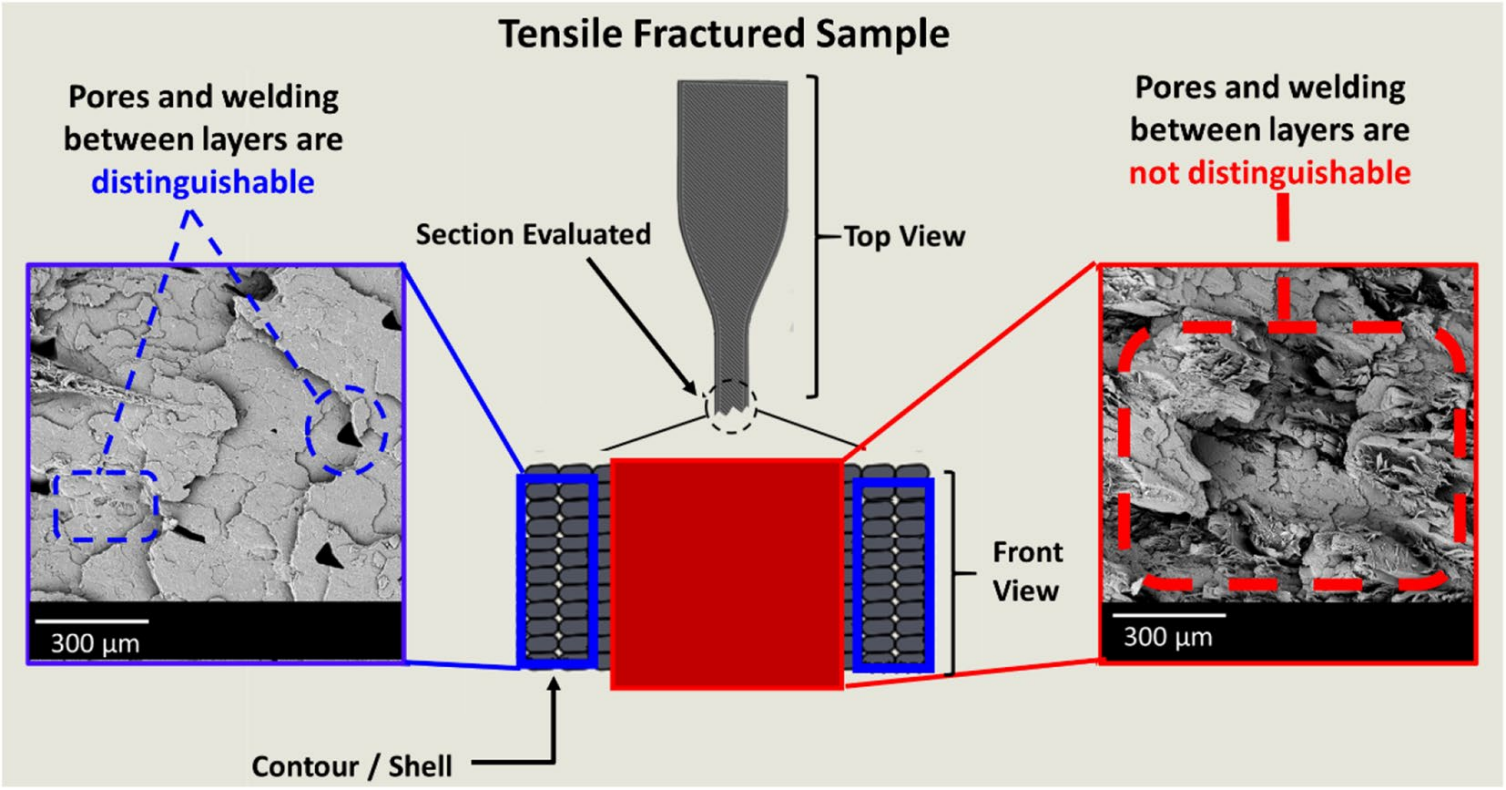
Schematic representation of a 3D printed tensile fractured sample. The surface morphology was evaluated in the blue sections since the bond formation between layers was not distinguishable in the red section. The figures were generated from SolidWorks 2016 and Phenom World-Phenom Prosuite.

A schematic representation of the tensile fractured morphology of 3D printed parts for PHBV:PLA:CE (40:60:0.25) wt% based blend is shown in Fig. 5. The middle section of the tensile fractured samples was not used to evaluate the diffusion-based fusion between layers after tensile failure since the morphological structure was not distinguishable due to the ± 45° raster angle pattern and brittle nature of the blends. Consequently, the product shell or contour, printed at 0° raster angle, was used to evaluate the structure integrity of the samples.

**Figure 6:**
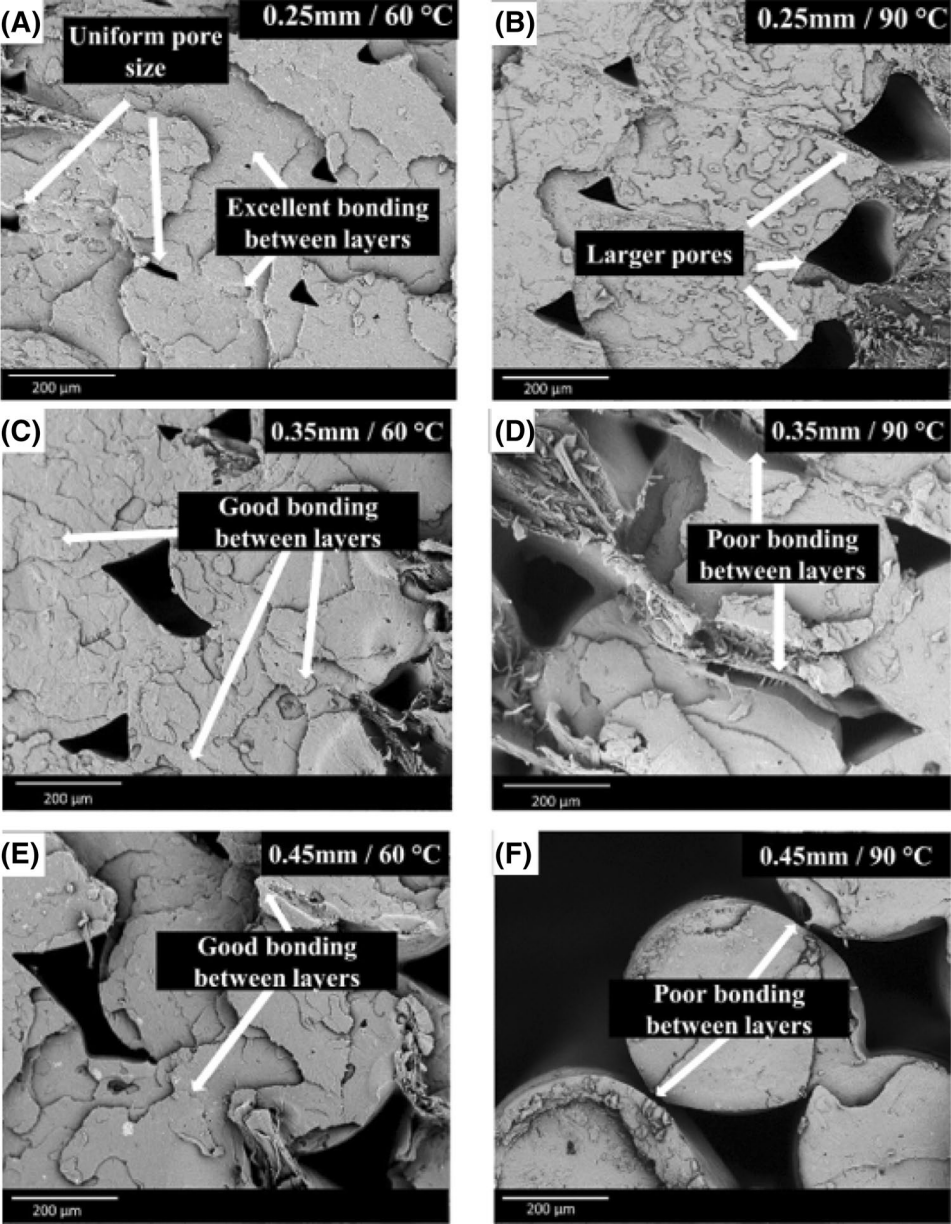
SEM images of tensile fractured PHBV:PLA:CE (40:60:0.25) 3D printed samples with (**A**) 0.25 mm layer thickness and bed temperature 60 °C, (**B**) 0.25 mm layer thickness and bed temperature 90 °C, (**C**) 0.35 mm layer thickness and bed temperature 60 °C (**D**) 0.25 mm layer thickness and bed temperature 90 °C (**E**) 0.45 mm layer thickness and bed temperature 60 °C, (**F**) 0.45 mm layer thickness and bed temperature 90 °C. The photos were generated from PhenomWorld-Phenom ProSuite.

In FFF, residual stresses and diffusion-based fusion are important factors involved in good bond formation between layers^7,15,35,36^. Samples with a layer thickness of 0.25 mm were found to have smaller and more uniform pores when compared to 0.35 mm and 0.45 mm layer thickness. For samples printed at a bed temperature of 60 °C, good bonding between layers was observed, as seen in Fig. 6A,C,E. On the other hand, using a bed temperature of 90 °C resulted in samples with poor weld formation between layers and enlarged pores after tensile failure resulting from the residual stress at higher bed temperature, as seen in Fig. 6B,D,F. In a study conducted by Wang and Gardner^7^, the layer thickness effect on interlayer bond formation was investigated using a 0° raster angle. They concluded that 3D printed samples with smaller layer thickness resulted in a higher degree of diffusion-based fusion between layers. In a study by Choi et al*.*^36^, it was observed that 3D printing of ABS using a temperature 20 to 30 °C higher than the glass transition temperature reduced the mechanical properties of the samples. In addition, Kantaros and Karaleka^15^ reported that the residual stress of 3D printed products could be improved by using smaller layer size. In the case of PHBV:PLA:CE (40:60:0.25) based blend, it is hypothesized that, due to the highly crystalline nature of PHBV, using a bed temperature closer to the crystallization temperature of PHBV may have resulted in faster crystallization, leading to unwanted residual stress.

The effects of CE on the morphology of PHBV:PLA:CE 3D printed samples was also investigated using scanning electron microscopy. The surface morphology of all the PHBV:PLA:CE blends can be seen in Supplementary Fig. S4. No clear distinction in the morphology of 3D printed samples was observed for any of the PHBV:PLA:CE based blends. Similar observations for PHBV:PLA based blends were reported by Gerard and Budtova^37^. A co-continuous morphology was characteristic as the PHBV:PLA blends approximated equal ratios^37^.

### Mechanical characterization of the 3D printed samples

**Figure 7:**
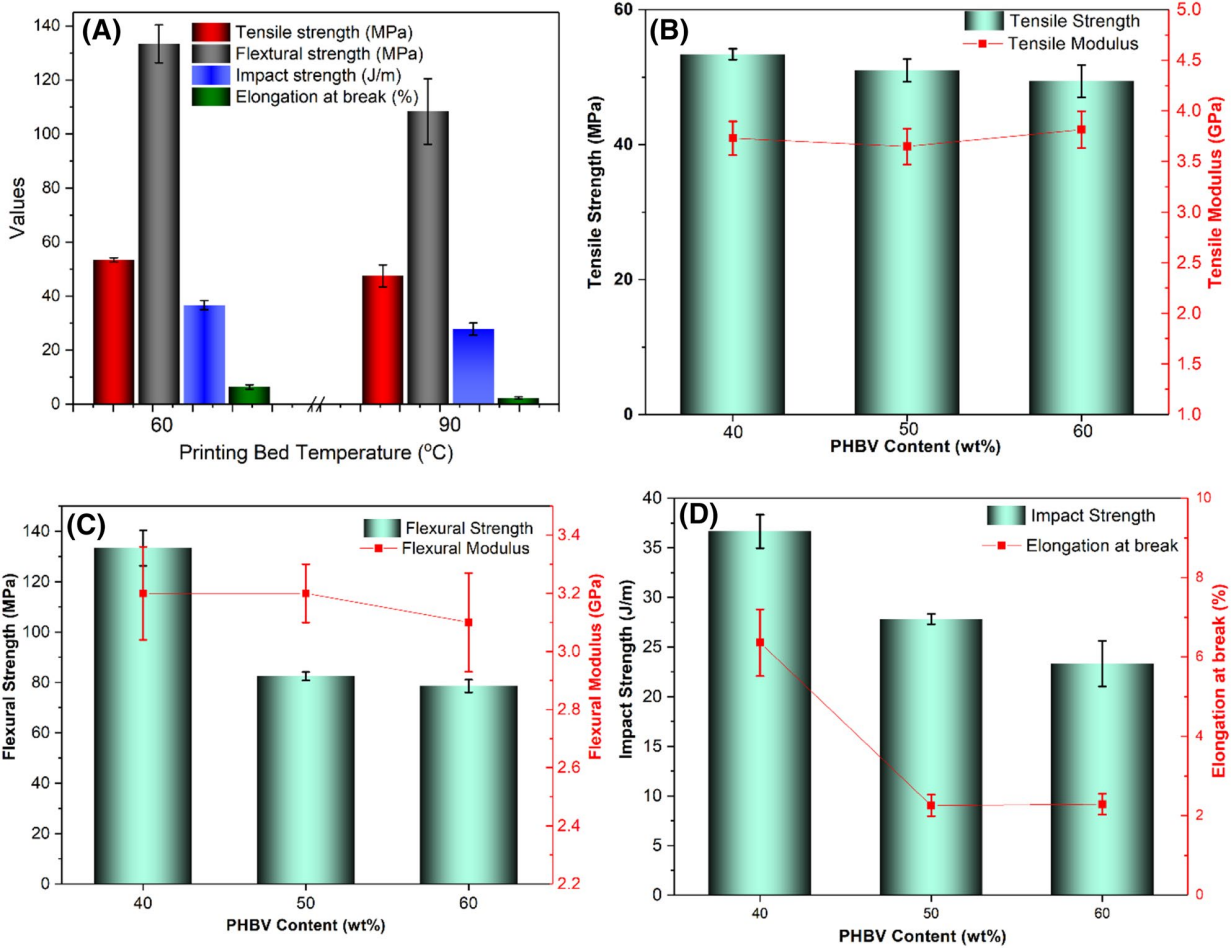
PHBV: PLA: CE based blends (**A**) the effect of printing temperature on the mechanical performance of PHBV:PLA:CE (40:60:0.25) printed with a thickness of 0.25 mm; (**B**) tensile strength and modulus, (**C**) flexural strength and modulus, and (**D**) impact strength and elongation at break (all the blends printed at bed temperature of 60 °C). The figures were generated from Origin 8, OriginLab.

For PHBV:PLA:CE (40:60:0.25) wt% based blend, better mechanical properties were obtained when using a bed temperature of 60 °C and a 0.25 mm layer thickness. The effects of bed temperature on the mechanical performance of the blends were given in Fig. 7A. Like the DMA characterizations, the stiffness and toughness of the samples printed with lower layer thickness (0.25 mm) were better as compared to the blends with higher layer thickness. This resulted from the following: (i) the sample printed with lower layer thickness possessed a smaller cell size between layers; (ii) samples with a layer thickness of 0.25 mm were found to have smaller and more uniform pores when compared to 0.35 mm and 0.45 mm. At same layer thickness of 0.25 mm, the tensile and flexural strength of the 3D printed blends were enhanced by 12% and 23%, respectively, when using a bed temperature of 60 °C rather than 90 °C. Also, the toughness of the sample printed at 60 °C was higher than that of sample at 90 °C, reflected by the 1.3 times higher impact strength and 2.8 times higher of elongation at break. This correlated with surface morphological results, and it was concluded that good bond formation between layers was crucial for the enhancement of mechanical properties.

To study the effects of PHBV contents on the mechanical properties of the blends, the PHBV:PLA:CE based blends printed at optimization conditions, i.e. a layer thickness of 0.25 mm and a bed temperature of 60 ˚C, were chosen by authors. The mechanical performances of the blends were characterized as seen in Fig. 7B–D. The tensile strength and modulus were independent of PHBV content because of the close crystallinity and morphology of these samples. The decreased flexural modulus of the blends with increasing PHBV is attributed to the lower flexural modulus of PHBV used in this research. However, the toughness including the impact strength and elongation at break are decreased with increasing PHBV mainly because of the higher brittleness of the PHBV compared to PLA. Overall, better mechanical properties were obtained for the PHBV:PLA:CE (40:60:0.25) wt% based blend. This improvement in mechanical properties with increasing PLA content was attributed to the toughening effect of PLA on PHBV, as previously reported by Nanda et al*.*^34^.

## Conclusion

The use of a chain extender (CE) successfully improved the filament fabrication and 3D printability of PHBV:PLA based blends. Differential scanning calorimetry analysis showed a decreasing trend in the degree of crystallinity of PHBV:PLA:CE blends with increasing PLA content, and was corroborated with the findings of the polarized optical microscopy that showed the local absence of birefringence in PHBV crystals. Rheological analysis served as a suitable indicator for the selection of an appropriate printing temperature. A printing temperature of 220 °C, printing speed of 45 mm s^−1^, bed temperature of 60 °C and layer thickness of 0.25 mm were found to be the optimal processing parameters to enhance mechanical performance of the products. This was confirmed by dynamic thermo-mechanical and mechanical analyses showing stiffer and stronger products under these conditions. The successful 3D printing of PHBV:PLA:CE based blends will help create sustainable alternatives and lower the dependence on petroleum-based 3D printing materials and expected to be used in biomedical applications.

## Experimental setup and methods

### Materials

The thermoplastic polyester poly(lactic acid) (PLA) Ingeo biopolymer 4043D, multi-purpose film grade, a product of NatureWorks, LLC, USA was used. The melting temperature (*T*_m_) range and melt flow index (MFI) of PLA were 145 to 160 °C and 6 g 10 min^−1^ (190 °C, 2.16 kg), respectively, as noted from the technical data sheet (TDS). Poly(3-hydroxybutyrate-*co*-3-hydroxyvalerate), reference ENMAT PHBV resin, Y1000P, was obtained from TianAn Biopolymer, China. The *T*_m_ and MFI of PHBV as noted from the TDS were 174 °C and 12.4 g 10 min^−1^ (180 °C, 2.16 kg), respectively. Joncryl grade ADR-4368C, an epoxy functionalized styrene-acrylate copolymer compatibilizer, a product of BASF, Ludwigshafen, Germany was used as a CE. Joncryl contained 285 g mol^−1^ reactive epoxy functional group according to the TDS provided by BASF Corporation, Germany.

### 3D printing filament preparation

Prior to mixing PLA and PHBV pellets with CE, both biopolymers were dried overnight in an oven at 70 °C. The CE flakes were ground using a mortar and pestle to form a powder. Subsequently, both PLA and PHBV pellets were mixed with CE powder at approximately 80 °C. The PHBV:PLA compatibilized blends were processed using a twin-screw extruder, Micro-27 from Leistritz, Germany. The length to diameter (L/D) ratio was 48. A gravimetric feeder was used as a feed mechanism and the material blends were processed at 180 °C in all zones with a screw speed of 100 rpm. The filaments were collected using an automated pelletizer without a cutting blade to create a monofilament and allowed to dry overnight at 70 °C before printing to avoid the hydrolysis during 3D printing. Pilot experimentation showed that at least 40% PLA was needed to improve the printability of PHBV. Therefore, three different blends of PHBV:PLA (60:40; 50:50; and 40:60) were used in this research.

### 3D printing

The 3D printing of parts was performed using a 3D desktop printer, LulzBot TAZ 6, manufactured and distributed by Aleph Object, Inc, USA. A single tool head extruder, LulzBot Tool Head v2.1, was used for the melting and deposition of the thermoplastics. A print surface, LulzBot TAZ Heat Bed, with a poly(etherimide) (PEI) sheet was used as the surface platform with a bed temperature range between 20 and 120 °C. A printing temperature of 220 °C, printing speed of 45 mm s^−1^ and infill density of 100% were kept constant for all experiments. The raster layer angle of the 3D printing samples was set as ± 45°. The layer thickness in the vertical or Z direction was adjusted accordingly to study the effects of thickness on the mechanical properties and surface finish of the 3D printed samples. Since Shubham et al*.*^6^ reported no improvement in mechanical properties for layer thickness below 0.2 mm, the layer thicknesses chosen for this research were 0.25 mm, 0.35 mm and 0.45 mm consisted of 13, 9 and 7 layers, respectively, in the vertical direction. Surface bed temperatures of 60 and 90 °C were selected to be above the glass transition temperature (*T*_g_) (58 to 59 °C) and best crystallization temperature of PLA. The previous research in our group by Benwood et al*.*^9^ found that tensile and impact strength of PLA were significantly improved at a bed temperature of 90 °C due to an increase in the crystallinity of PLA. For each experiment, one sample was printed at a time and removed after the surface bed temperature reached 50 °C or less for easy removal of the flexural, tensile and impact samples. Table 2 shows the list of 3D printing parameters.

**Table 2:** Summary of 3D printing parameters used for the 3D printing of PHBV:PLA:CE (40:60:0.25) wt% based blend.

3D printing parameters	Value				Unit
Printing temperature	220				°C
Layer thickness	0.25	0.35		0.45	mm
Printing speed	45				mm s^−1^
Bed temperature	60		90		°C
Infill	100				%
Raster angle	± 45				°

### Characterization

#### Differential scanning calorimetry (DSC)

A TA instruments Inc. Q200 calorimeter was used to measure the *T*_g_ and *T*_m_ under a nitrogen atmosphere with a flow rate of 50 L min^−1^. For each blend, samples of approximately 11 mg were collected and measured using a micro balance, Mettler Toledo Model XP6, Switzerland, and encapsulated in sealed aluminum pans. The samples were analysed using a heat-cool-heat ramp cycle rate of 10 °C min^−1^. The temperature profile consisted of a heating ramp from − 20 to 200 °C, followed by cooling to − 20 °C and heating to 200 °C. The data was recorded and analysed using Universal Analysis 2000 software (TA Instruments, USA). The percent crystallinity was calculated using Eq. 1^19^:

1 $$ Crystallinity\, \left( \% \right) = X_{c} = \frac{{\Delta H_{m} - \Delta H_{cc} }}{{\Delta H_{m}^{0} \times W}} \times 100\% $$

where $\Delta H_{cc}$ is the measured enthalpy of cold crystallization, $\Delta H_{m}$ is the measured enthalpy, *W* is the weight fraction of the polymer in the blend system and $\Delta H_{m}^{0}$ is the theoretical melting enthalpy (100%). The $\Delta H_{m}^{0}$ values for PHBV and PLA obtained from literature were 146 J g^−1^^38^ and 93.7 J g^−1^^39^, respectively.

#### Polarized optical microscopy (POM)

A polarized optical microscope, ECLIPSE LV100, manufactured by Nikon (Minato, Japan) and equipped with a Linkam hot stage was used to observe the size of the spherulites under controlled heating and cooling. Prior to the optical microscopy analysis, the samples were prepared by melting at 180 °C and gently manually pressed between two layers of glass to obtain a thin film. The parameters used to prepare the samples were selected to observe the formation of crystals using similar conditions as applied in 3D printing. The prepared samples were placed on a hot stage and heated at 50 °C min^−1^ and held at 200 °C for 2 min to melt the crystals and remove any thermal history, followed by rapid cooling at 50 °C min^−1^ and isothermal treatment at 60 °C for 30 min. Magnifications of 10 and 20 times were used.

#### Viscosity and morphology of materials

Rheological characterization was performed using a MCR 302 rheometer from Anton Paar GmbH, Graz, Austria. The 3D printed samples in ± 45° were used for rheological testing. The complex viscosity was measured using parallel plates with a gap between plates of 1.0 mm and a range of angular frequency between 0.01 to 100 rad/s. All measurements were conducted at temperatures ranging from 180 to 230 °C under a nitrogen atmosphere. This range of temperature was used to create a time temperature superposition (TTS) master curve, which was used to shift frequency data to calculate the complex viscosity of the PHBV:PLA based blends during 3D printing. The horizontal shift factor was calculated using RheometerPass software and the Williams-Landel-Ferry model with a ± 2% frequency accuracy. The shear stress experienced by the filament during extrusion was estimated using Eq. 2^13^:

2 $$ \dot{\gamma } = \frac{{8\overline{\upsilon }}}{D} $$

where $\dot{\gamma }$ represents the shear rate, $\overline{\upsilon }$ represents the extrusion rate and *D* the nozzle diameter. The nozzle diameter of the 3D printer was 0.5 mm. A shear rate of 720 s^−1^ was estimated during the 3D printing of samples at a printing speed of 45 mm s^−1^.

A scanning electron microscope, Phenom ProX, ATA Scientific Instruments (Taren Point, Australia), was used to study the surface morphology of the specimens. To observe the morphology of the blends, 3D printed samples were cryofractured using liquid nitrogen in order to obtain a surface without plastic deformation. To observe the interlayer bonding between layers, tensile samples were analyzed after failure. For both analyses, a thin gold layer was used to coat the surface for 10 s prior to being examined with back scattering electrons at 10 kV acceleration voltage.

#### Mechanical testing

The Izod samples were notched using a motorized notching cutter from Testing Machine Inc. TMI, USA, and the impact strength was measured using a ZwickRoell Model HIT25P, Germany, with a 2.75 J pendulum hammer according to ASTM D256. Tensile and flexural properties were measured according to ASTM D638 and ASTM D790, respectively, on an Instron 3382 Universal Testing Machine. The data were analysed using Blue Hill software.

#### Dynamic mechanical analysis (DMA)

A dynamic mechanical analyser, TA Instruments, New Castle, DE was used to measure the viscoelastic properties of rectangular 3D printed samples with dimensions of approximately 3.25 $\times$ 12.7 $\times$ 50 mm. The loss modulus, storage modulus and tan delta were determined using the dual cantilever measurement mode. The test parameters used were frequency and amplitude of 1 Hz and 15 μm, respectively, and a temperature range from − 20 to 120 °C at a rate of 3 °C min^−1^.

## Supplementary information

Supplementary Information
