# Supplementary material for: Study on the 3D printability of poly(3-hydroxybutyrate-co-3-hydroxyvalerate)/poly(lactic acid) blends with chain extender using fused filament fabrication
Source: Sci Rep. 2020 Jul 16;10:11804. doi: 10.1038/s41598-020-68331-5 (PMC7367353; doi:10.1038/s41598-020-68331-5)
Supplement: Supplementary file 1 — Supplementary Information [file 41598_2020_68331_MOESM1_ESM.docx]

Study on the 3D printability of poly(3-hydroxybutyrate-*co*-3-hydroxyvalerate)/ poly(lactic acid) blends with chain extender using fused filament fabrication

*Miguel A. Vigil Fuentes^a,b^, Suman Thakur^a^, Feng Wu^a^ , Manjusri Misra^a, b, *^, Stefano Gregori^a,b^ Amar K. Mohanty^a, b,*^*

^a^Bioproducts Discovery & Development Centre, Department of Plant Agriculture, Crop Science building, University of Guelph Ontario, Canada

^b^School of Engineering, Thornbrough building, University of Guelph, Ontario, Canada

Corresponding authors:

*E mail: M. Misra: [mmisra@uoguelph.ca](mailto:mmisra@uoguelph.ca)

*E mail: A. K. Mohanty: [mohanty@uoguelph.ca](mailto:mohanty@uoguelph.ca)

Supplementary information

**
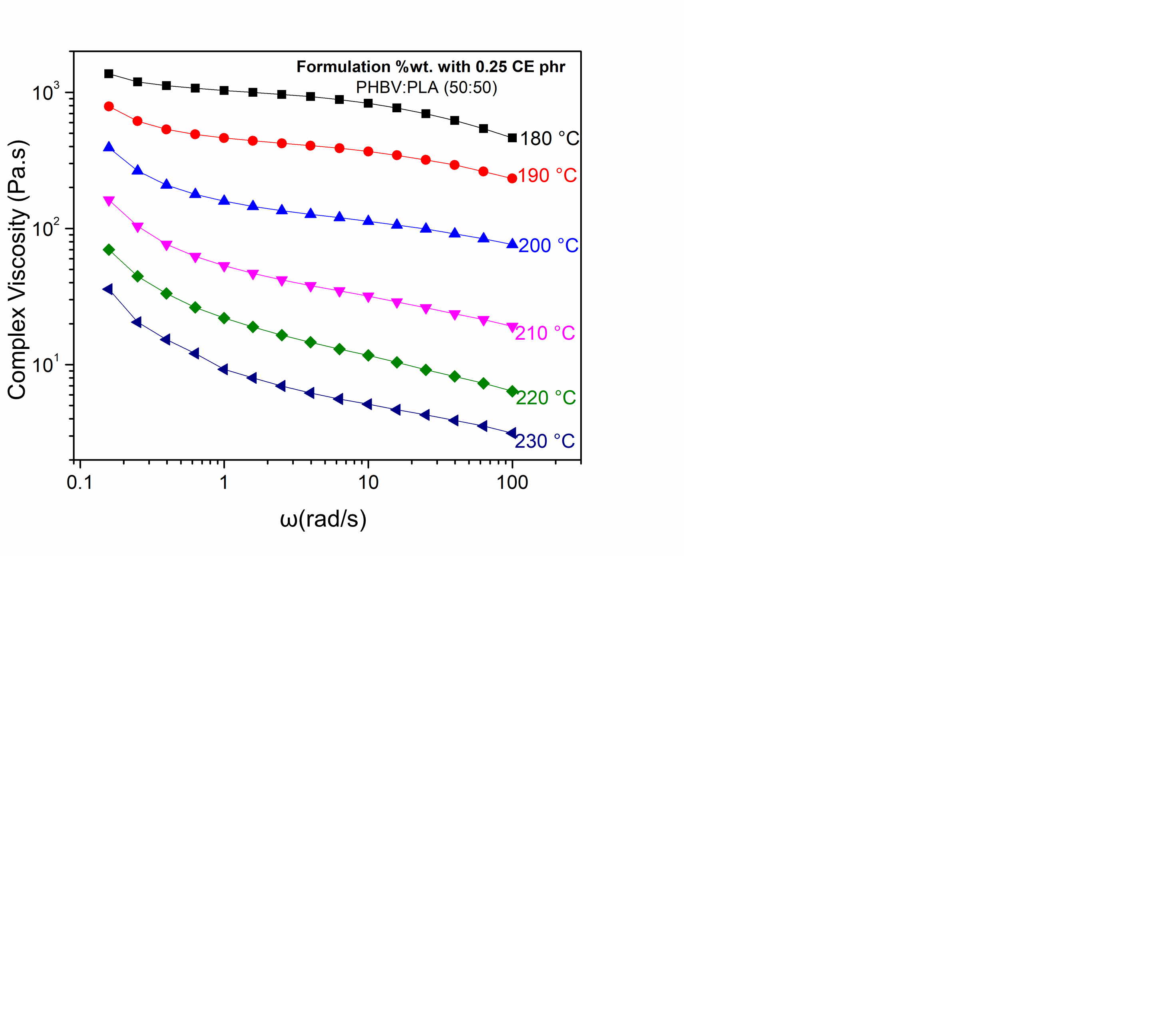

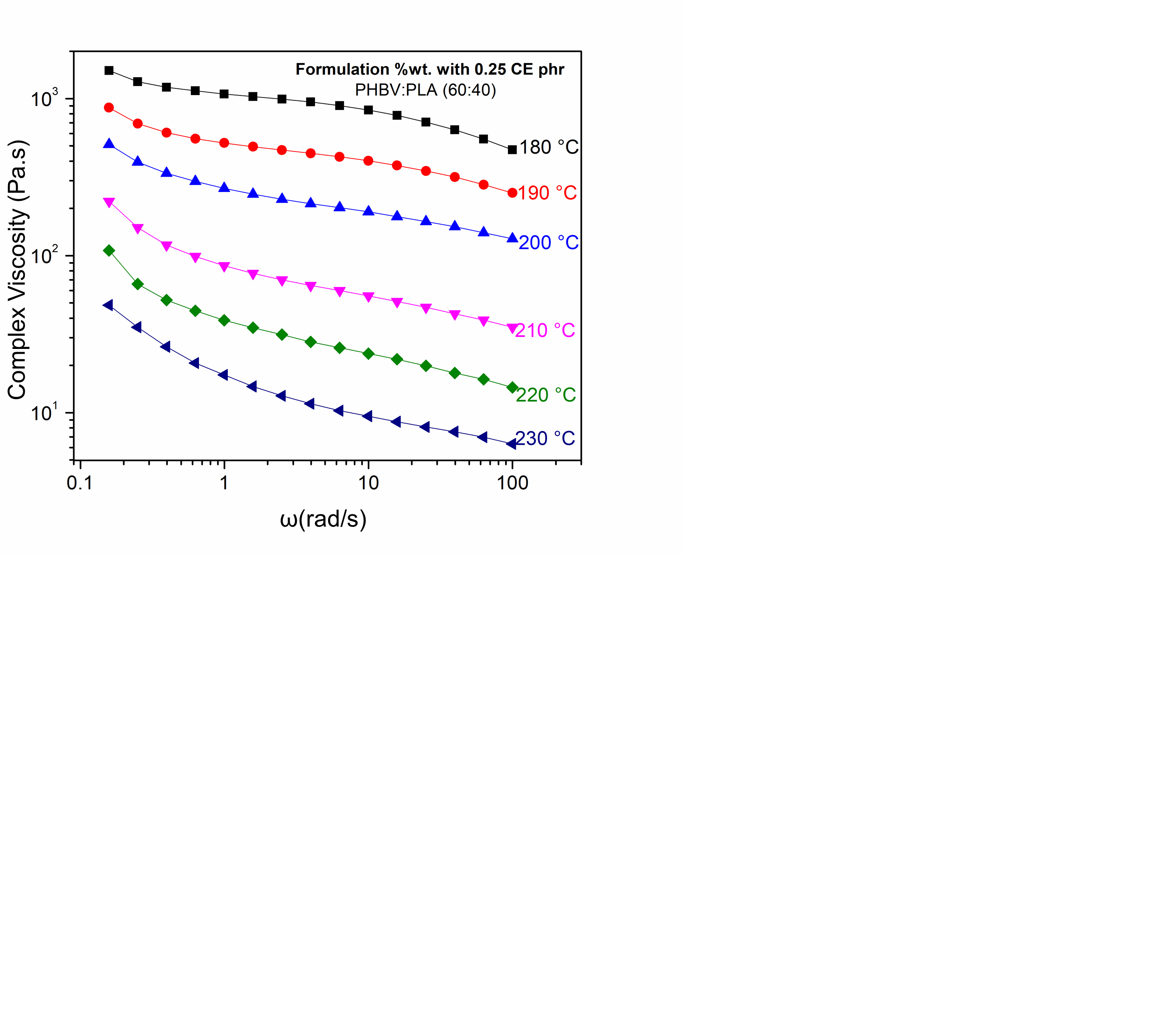
**

**(A)**

**(B)**

**Figure S1:** complex viscosity of the (A) PHBV:PLA:CE (50:50:0.25) and (B) PHBV:PLA:CE (60:40:0.25) wt.% based blend using temperature profiles between 180 and 230 °C. The figures were generated from Origin 8, OriginLab.


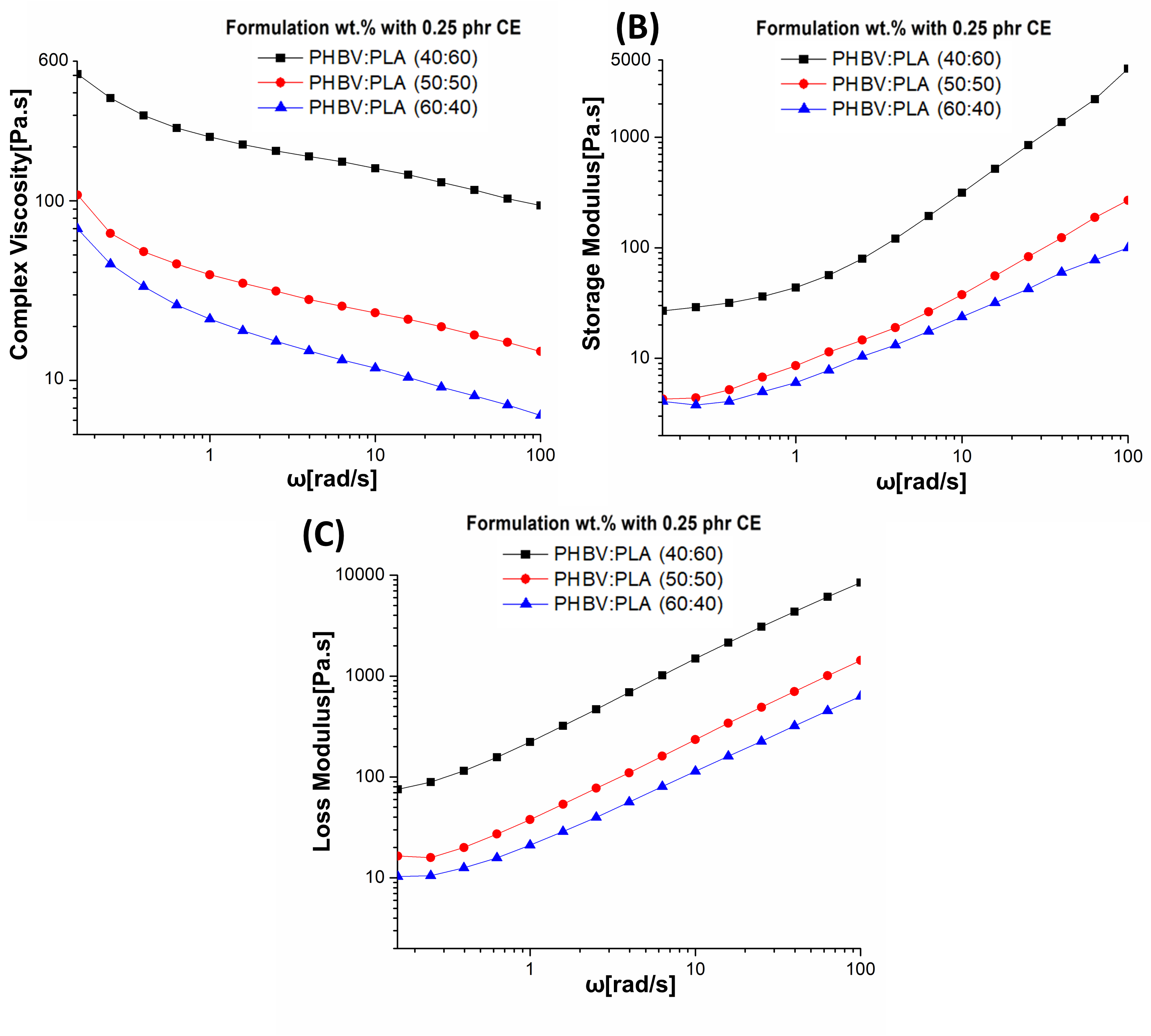


**(A)**

**Figure S2:** (A) complex viscosity, (B) storage modulus, and (C) loss modulus of PHBV:PLA:CE (40:60:0.25), (50:50:0.25) and (60:40:0.25) wt.% based blends at 220 ºC. The figures were generated from Origin 8, OriginLab.

**
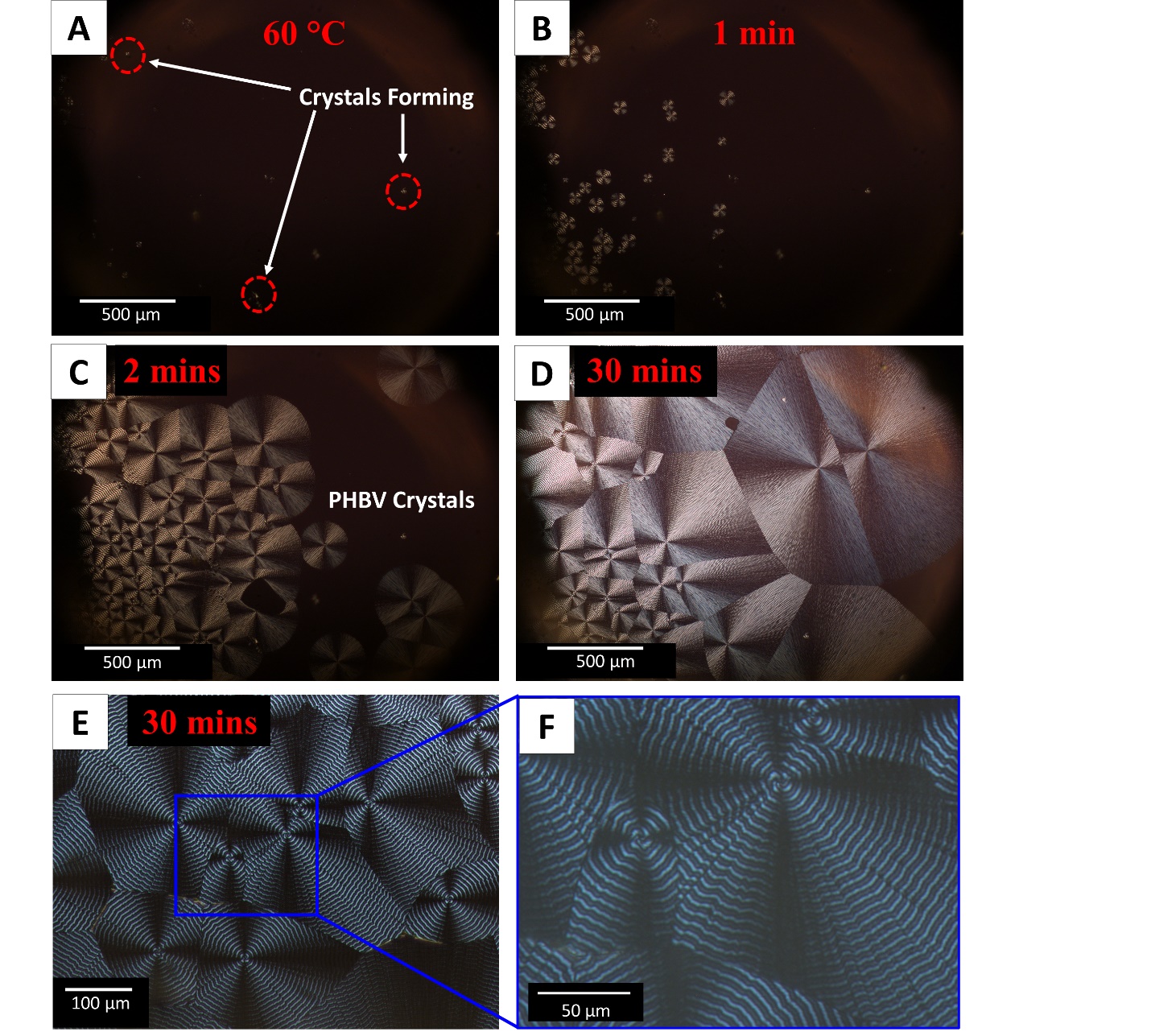
**

**Figure S3:** Crystal morphology of PHBV at 60 °C (A) when crystals PHBV began forming, (B) after 1 minute, (C) after 2 minutes, (D) after 30 minutes, (E) magnified image of crystals after 30 minutes and (F) magnified part of E. The photos were generated from NIS-Elements BR3.2 64-bit.

**
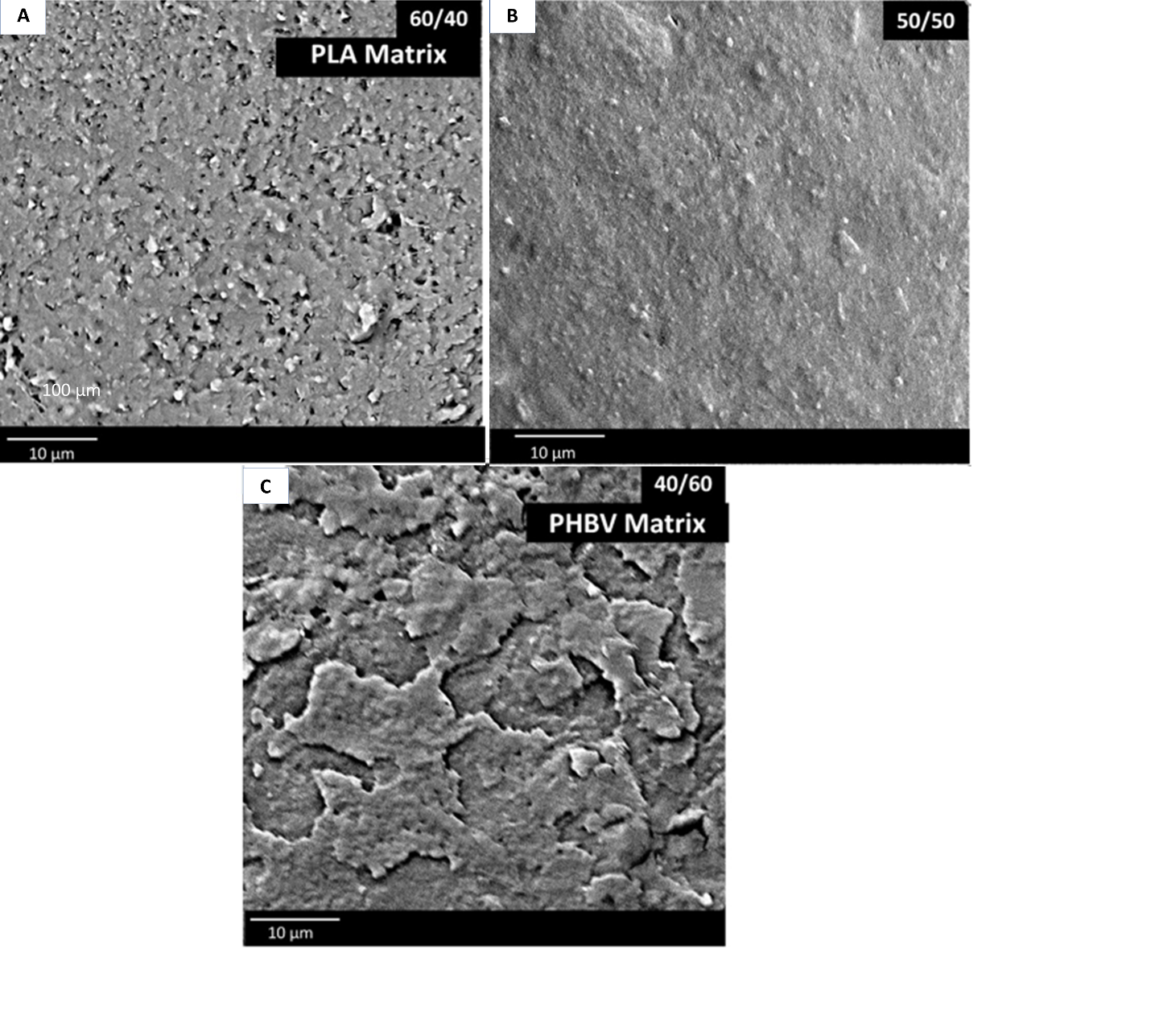
**

**Figure S4:** Surface morphology of 3D printed samples for PHBV:PLA:CE based blends (A) ratio 40:60:0.25 wt.%, (B) 50:50:0.25 wt.%, and (C) 60:40:0.25 wt.%. The photos were generated from Software: PhenomWorld-Phenom ProSuite
